# Comparison of Shedding Characteristics of Seasonal Influenza Virus (Sub)Types and Influenza A(H1N1)pdm09; Germany, 2007–2011

**DOI:** 10.1371/journal.pone.0051653

**Published:** 2012-12-11

**Authors:** Thorsten Suess, Cornelius Remschmidt, Susanne B. Schink, Brunhilde Schweiger, Alla Heider, Jeanette Milde, Andreas Nitsche, Kati Schroeder, Joerg Doellinger, Christian Braun, Walter Haas, Gérard Krause, Udo Buchholz

**Affiliations:** 1 Department of Infectious Disease Epidemiology, Robert Koch Institute, Berlin, Germany; 2 National Reference Centre for Influenza, Robert Koch Institute, Berlin, Germany; 3 Centre for Biological Security, Division of Highly-Pathogenic Viruses (ZBS1), Robert Koch Institute, Berlin, Germany; University of Hong Kong, Hong Kong

## Abstract

**Background:**

Influenza viral shedding studies provide fundamental information for preventive strategies and modelling exercises. We conducted a prospective household study to investigate viral shedding in seasonal and pandemic influenza between 2007 and 2011 in Berlin and Munich, Germany.

**Methods:**

Study physicians recruited index patients and their household members. Serial nasal specimens were obtained from all household members over at least eight days and tested quantitatively by qRT-PCR for the influenza virus (sub)type of the index patient. A subset of samples was also tested by viral culture. Symptoms were recorded daily.

**Results:**

We recruited 122 index patients and 320 household contacts, of which 67 became secondary household cases. Among all 189 influenza cases, 12 were infected with seasonal/prepandemic influenza A(H1N1), 19 with A(H3N2), 60 with influenza B, and 98 with A(H1N1)pdm09. Nine (14%) of 65 non-vaccinated secondary cases were asymptomatic/subclinical (0 (0%) of 21 children, 9 (21%) of 44 adults; p = 0.03). Viral load among patients with influenza-like illness (ILI) peaked on illness days 1, 2 or 3 for all (sub)types and declined steadily until days 7–9. Clinical symptom scores roughly paralleled viral shedding dynamics. On the first day prior to symptom onset 30% (12/40) of specimens were positive. Viral load in 6 asymptomatic/subclinical patients was similar to that in ILI-patients. Duration of infectiousness as measured by viral culture lasted approximately until illness days 4–6. Viral load did not seem to be influenced by antiviral therapy, age or vaccination status.

**Conclusion:**

Asymptomatic/subclinical infections occur infrequently, but may be associated with substantial amounts of viral shedding. Presymptomatic shedding may arise in one third of cases, and shedding characteristics appear to be independent of (seasonal or pandemic) (sub)type, age, antiviral therapy or vaccination; however the power to find moderate differences was limited.

## Introduction

Influenza viral shedding studies are important as they help to understand the epidemiology of the virus and form the basis for rational preventive strategies. Among the parameters that can be obtained from viral shedding studies are the degree of viral shedding before/after symptom onset, duration of viral shedding, course of clinical symptoms, serial interval, age dependency of the shedding profile and proportion of asymptomatic/subclinical infections. Furthermore, results such as incubation period or latency period can be used for the parameterization of modelling studies on population level[1]–[3]. Finally, mathematical manipulation of data can aid to estimate the basic reproduction number, the recovery rate or the proportion of transmission events prior to symptoms [4].

Before the influenza A(H1N1)pdm09 pandemic volunteer challenge studies with seasonal virus (sub)types had been conducted and were summarized in a meta-analysis by Carrat et al. in 2008 [5]. On the other hand studies investigating shedding properties of the A(H1N1)pdm09 virus have been based on naturally occurring infections, therefore limiting comparability. To date only two studies have compared shedding and symptom characteristics of the pandemic A(H1N1)pdm09 virus with seasonal, pre-pandemic influenza virus [6], [7]. However, both studies did not include shedding dynamics of influenza B. In addition, although both have addressed asymptomatic and presymptomatic shedding the number of individuals where these aspects could be investigated was limited.

We conducted a prospective household based study of seasonal (A(H3N2), A(H1N1), B), and pandemic (A(H1N1)pdm09) influenza during four consecutive influenza seasons from 2007/2008 to 2010/2011 in Berlin and Munich, Germany. Objectives were to describe shedding dynamics and course of illness by (sub)type and age, to compare shedding dynamics in asymptomatic (subclinical) vs. symptomatic patients, in vaccinated vs. non-vaccinated patients, as well as in those treated with oseltamivir vs. those not treated, and to describe the relationship of molecular viral load with results using tissue-culture methods.

## Methods

### Recruitment and Follow-up of Participants

From general practitioners and pediatricians in Berlin and Munich (Germany) we recruited influenza households during four consecutive influenza seasons: January 2008-April 2008 (season 1 - dominated by seasonal/prepandemic influenza A(H1N1) and influenza B), January 2009-April 2009 (season 2 - dominated by influenza A(H3N2) and influenza B), November 2009-January 2010 (season 3 - dominated by influenza A(H1N1)pdm09), and January-April 2011 (season 4 - dominated by influenza A(H1N1)pdm09 and influenza B). Munich was a study site only in seasons 1 and 2. During seasons 3 and 4, the study was embedded in a cluster randomised trial on the effectiveness of facemasks and hand hygiene to reduce influenza transmission in households [8].

Household index patients eligible for inclusion had to have influenza-like illness (defined as fever and [cough or sore throat]) and to present at the recruiting physician within two days of symptom onset, had to have a positive rapid antigen test for influenza (later to be confirmed by quantitative reverse transcription polymerase chain reaction [qRT-PCR]), and had to be more than two years of age. Exclusion criteria were pregnancy, severely reduced health status, HIV infection, and belonging to a single person household.

We obtained written informed consent from all study participants. If these were less than 18 years of age we asked their parents or legal guardians to provide proxy written consent, with additional written consent from those participants aged 14 to 17 years of age.

The observation period for each household lasted 8 days, starting on the day of symptom onset or up to two days after symptom onset of the index patient counting the day of symptom onset as day 1 for the household. We collected specimens and data from all household members. Household visits were scheduled daily during the observation period of seasons 1 and 2 and on days 2, 3, 4, 6, and 8 (five times) or on days 3, 4, 6, and 8 (four times) depending on the day of recruitment in seasons 3 and 4. If one of the household contacts developed symptoms (fever/chills, cough or sore throat) and was laboratory confirmed as a secondary case two further visits were scheduled for days 10 and 12.

### Specimen Collection

During all household visits we obtained nasal wash specimens (or – if these were not possible – nasal swabs) from all participating household members. For the collection of nasal wash, we used 5 mL of isotonic saline, which were instilled into one nostril with participant’s heads tilted backwards. Participants were asked to remain in this position for 10–15 s while making hard ‘K’ sounds without swallowing. Subsequently, the participants were told to tilt their heads forward and the fluid was collected in a sterile cup [9]. Nasal swabs were collected by using virus transport swabs (Mastaswab™; MAST Diagnostica, Reinfeld, Germany). A subgroup of participants (aged 14 years or older) in seasons 1 and 2 agreed to provide blood samples (a maximum of five EDTA samples per participant collected before symptom onset, on the day of symptom onset, and on the first three after symptom onset). Additionally in seasons 1 and 2, index patients were asked to provide one stool sample on one of the first three illness days. Samples were stored refrigerated (at a temperature of approximately 5°C) before analysis with the exception of samples tested by viral culture which were stored frozen (at approximately −80°C) before analysis (see below) [10].

After the first household visit, all participants self-recorded symptoms (fever, shivering, measured temperature, cough, sore throat) in a daily monitoring questionnaire. We defined children as persons aged less than 14 years, adults were at least 14 years old. “Timely antiviral therapy” (if prescribed by their physician) started within two days of symptom onset. We defined a symptomatic secondary influenza virus infection as a laboratory confirmed influenza infection in a household member who developed fever (>38.0°C), cough, or sore throat in the observation period. We termed all other secondary cases as asymptomatic/subclinical.

For the assessment of viral shedding profiles among symptomatic patients we used as the day of symptom onset the day when the patient had fever, cough or sore throat for the first time. If the last available specimen of a participant was negative we assumed that viral shedding had ceased. To assess viral shedding in asymptomatic/subclinical individuals we restricted analysis to those who had two negative samples before the first positive sample and who had at least one further sample taken after the first positive sample. To exclude the possibility that this positive sample was taken on the first presymptomatic day (i.e. followed by onset of symptoms on the next day) we assured that no fever, cough or sore throat was present on the day (or any other day) after the day of the first positive sample. To describe the course of illness we calculated a daily symptom severity score on a four level scale from 0 (not present) to 3 (severe) for each of the following symptoms: fever/chills, cough, and sore throat. Thus the daily symptom score ranged from 0 points (no symptoms) to 9 points (all symptoms with maximum severity).

### Laboratory Analysis

Nasal wash specimens were analysed by qRT-PCR at the Centre for Biological Security, Unit for Highly-Pathogenic Viruses (season 3) and the National Reference Centre for Influenza (seasons 1, 2 and 4) both located at the Robert Koch Institute in Berlin, Germany. Nasal swabs were taken up by 3 mL of Minimum Essential Medium Eagle containing 10,000 U/ml of streptomycin/penicillin and afterwards treated just like nasal wash specimens. RNA was extracted using either the MagNA Pure 96 DNA and Viral Nucleic Acid Small Volume Kit (Roche Applied Science, Mannheim, Germany) on MagNA Pure 96 instrument (Roche Applied Science) or the MagAttract Viral RNA M48 Kit (Qiagen GmbH, Hilden, Germany) according to the manufacturer’s suggestions. Details about the PCR protocol as well as primer and probe sequences have been published elsewhere [11]. Quantitative results were expressed as log of RNA copies/ml (log Copies/ml). A convenience sample of specimens from seasons 1 and 2 was also tested for quantification of infectious viruses by using a plaque assay in MDCK cell culture monolayer. The results are presented as plaque-forming units (PFU)/ml.

### Statistical Analysis

We defined the serial interval as the number of days between symptom onset of the household index case and symptom onset of the first secondary case. Other secondary household cases were only included in the calculation if their symptom onset occurred on the same day as the first secondary household case. Further household cases were not taken into account for the calculation of the serial interval because it was not possible to decide if they were secondary or in fact tertiary cases.

For descriptive analysis we used Wilcoxon’s ranksum test for numerical and chi-square-tests for categorical variables. For all analyses, we used two-sided tests and considered p-values of <0.05 as statistically significant. We performed analyses with Stata software, version 11 (Stata Corporation, Texas, USA).

### Ethics Statement

The Ethics Committee of the Charité Universitätsmedizin Berlin approved of the study (EA1/043/07).

## Results

### Study Population

**Figure 1:**
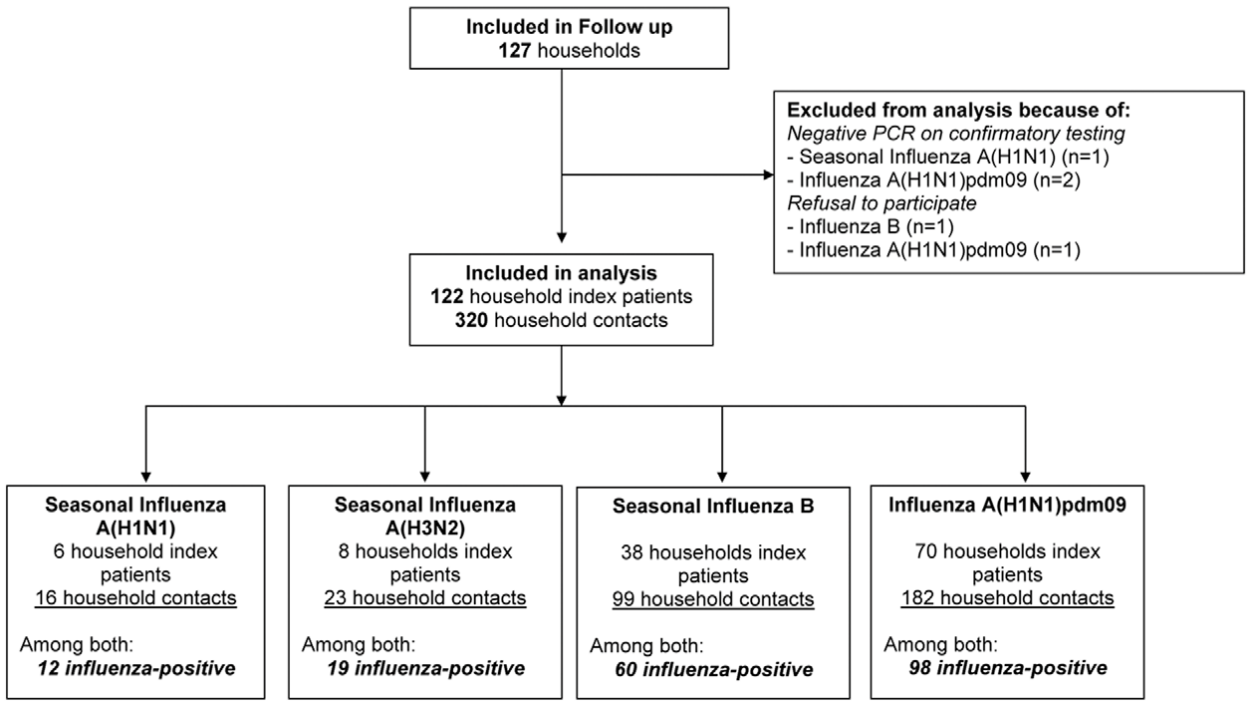
Study flow diagram.

We recruited 127 households over the four study periods. Five households had to be excluded from analysis because the initial rapid antigen test could not be confirmed by RT-PCR or the participants declined further participation, respectively (figure 1). The remaining 122 households comprised 442 participants (122 index patients and 320 household contacts). The index patient of six households (22 participants) was infected with seasonal/prepandemic influenza A(H1N1) infection (recruited in season 1), of eight households (31 participants) with influenza A(H3N2) (season 2), of 38 households (137 participants) with influenza B (seasons 1, 2, and 4) and of 70 households (252 participants) with influenza A(H1N1)pdm09 (seasons 3 and 4). This resulted in 12 laboratory confirmed cases of seasonal/prepandemic influenza A(H1N1)(6 index, 6 household contacts), in 19 laboratory confirmed cases of influenza A(H3N2) (8 index, 11 household contacts), in 60 laboratory confirmed cases of influenza B (38 index, 22 household contacts), and in 98 laboratory confirmed cases of influenza A(H1N1)pdm09 (70 index, 28 household contacts).

### Baseline Characteristics

Table 1 shows the baseline characteristics of index patients and household contacts stratified by influenza virus (sub)type of the household. In all study seasons index patients were almost exclusively children, while most household contacts were adults. The proportion of vaccinated household contacts differed significantly between (sub)types (p = 0.03, table 1), with the highest proportion observed in seasonal/prepandemic influenza A(H1N1) households (13%) and the lowest in A(H1N1)pdm09 (3%) households. Sixty-seven household contacts contracted influenza. Among 20 vaccinated household contacts two (10%) became laboratory confirmed cases (both A(H1N1)pdm09, both symptomatic), while among 300 non-vaccinated contacts 65 (22%) were laboratory confirmed cases, of whom 56 (86%) were symptomatic and 9 (14%) asymptomatic.

**Table 1:** Baseline characteristics of index patients and household contacts.

Variable	All (sub)types	Seasonal/prepandemic influenza A(H1N1)	SeasonalInfluenzaA(H3N2)	Seasonal influenza B	Influenza A(H1N1) pdm09	p-value
**Index Cases – n**	**122**	**6**	**8**	**38**	**70**	
Age (years) – median (IQR)	8.1 (6.0)	6.5 (5–12)	6.5 (2–11)	7 (5–10)	8 (5–10)	
Age<14 years – n/n (%)	113/122 (93)	5/6 (83)	7/8 (88)	35/38 (92)	66/70 (94)	0.7
Sex, male – n/n (%)	72/122 (59)	4/6 (67)	4/8 (50)	24/38 (63)	40/70 (57)	0.9
Vaccinated* – n/n (%)	5/122 (4)	1/6 (17)	0/8	3/38 (8)	1/70 (1)	0.1
Antiviral Therapy** – n/n (%)	41/122 (34)	2/6 (33)	0/8	15/38 (40)	24/70 (34)	0.2
Symptoms:						
ILI*** – n/n (%)	112/122 (92)	6/6 (100)	8/8 (100)	33/38 (87)	65/70 (93)	0.5
Fever/Chills – n/n (%)	122/122 (100)	6/6 (100)	8/8 (100)	38/38 (100)	70/70 (100)	
Cough – n/n (%)	113/122 (93)	5/6 (83)	8/8 (100)	34/38 (90)	66/70 (94)	0.5
Sore Throat – n/n (%)	77/122 (63)	4/6 (67)	7/8 (88)	28/38 (74)	38/70 (54.)	0.1
Myalgia – n/n (%)	103/122 (84)	6/6 (100)	7/8 (88)	33/38 (87)	57/70 (81)	0.6
**Household Contacts – n**	**320**	**16**	**23**	**99**	**182**	
Age (years) – median (IQR)	29.3 (15.0)	31.5 (9–41)	27 (26–39)	33 (12–41)	34.5 (17–42)	
Age<14 years – n/n (%)	76/320 (24)	6/16 (38)	5/23 (22)	28/99 (28)	37/182 (20)	0.3
Sex, male – n/n (%)	149/320 (47)	8/16 (56)	8/23 (35)	49/99 (50)	84/182 (46)	0.6
Vaccinated* – n/n (%)	20/320 (6)	2/16 (13)	1/23 (4)	11/99 (11)	6/182 (3)	0.03
**Influenza-positive household contacts - n**	**67**	**6**	**11**	**22** ##	**28** ##	
Vaccinated* – n/n (%)	2/67 (3)	0/6	0/11	0/22	2/28 (7)	0.7
Antiviral Therapy** – n/n (%)	8/67 (12)	1/6 (17)	2/11 (18)	4/22 (18)	1/28 (4)	0.2
Symptoms (among non-vaccinated secondary cases):						
ILI*** – n/n (%)	41/65 (63)	5/6 (83)	7/11 (64)	13/22 (59)	16/26 (62)	0.7
Fever/Chills – n/n (%)	44/65 (68)	5/6 (83)	7/11 (64)	14/22 (64)	18/26 (69.0)	0.8
Cough – n/n (%)	53/65 (82)	6/6 (100)	9/11 (82)	17/22 (73)	21/26 (81)	0.7
Sore Throat – n/n (%)	38/65 (59)	2/6 (33)	8/11 (73)	12/22 (55)	16/26 (62)	0.5
Myalgia – n/n (%)	50/65 (77)	4/6 (67)	9/11 (81)	15/22 (68)	22/26 (85)	0.4
Asymptomatic# – n/n (%)	9/65 (14)	0/6 (0)	2/11 (18)	4/22 (18)	3/26 (12)	0.7

* Vaccination defined as vaccination against pandemic influenza in season 2009/10 and trivalent seasonal vaccine in 2007/08, 2008/09 & 2010/11, at least 14 days before symptom onset in index patient or secondary household case. ** Antiviral therapy defined as treatment with oseltamivir or zanamivir within 2 days of symptom onset. *** ILI = Influenza-like-illness.

# Asymptomatic = no fever, no cough, no sore throat.

## Includes participants from all three intervention groups in seasons 3 and 4.

The overall proportion of asymptomatic/subclinical cases among non-vaccinated secondary cases (9/65; 14%) was dependent on age: while none of 21 non-vaccinated child cases were asymptomatic, 21% (9/44) of non-vaccinated adult cases were asymptomatic/subclinical (p = 0.03). Other variables such as sex, vaccination or antiviral therapy of the infecting index patient did not differ significantly between symptomatic and asymptomatic/subclinical secondary cases.

Symptoms of index patients (n = 122) as well as non-vaccinated secondary cases (n = 65) did not differ significantly between the virus (sub)types. 62% of A(H1N1)pdm09 non-vaccinated secondary household cases had symptoms consistent with ILI, similar to that of non-vaccinated A(H3N2) secondary cases (64%) and non-vaccinated secondary influenza B cases (59%), but lower than non-vaccinated secondary seasonal/prepandemic influenza A(H1N1) cases (83%). Differences among the groups were not statistically significant (p = 0.7).

### Serial Interval

On the basis of five secondary cases the mean serial interval for seasonal/prepandemic influenza A(H1N1) was estimated at 2.4±2.1 days (mean, standard deviation). For seasonal influenza A(H3N2), influenza B and influenza A(H1N1)pdm09 the mean serial intervals were 1.9±0.7 days (based on 7 secondary cases), 2.4±1.5 days (based on 14 secondary cases) and 2.4±1.5 days (based on 19 cases), respectively.

### Shedding Analyses

#### Blood and stool samples

In seasons 1 and 2, we obtained a total of 18 EDTA blood samples from four laboratory confirmed cases – all collected from symptomatic secondary household cases before and during the first three days after symptom onset. All of them were secondary household cases. Results for all samples were qRT-PCR negative. Seven index patients provided a total of seven stool samples. Results of qRT-PCR were also negative for these samples.

#### Shedding curves

**Figure 2:**
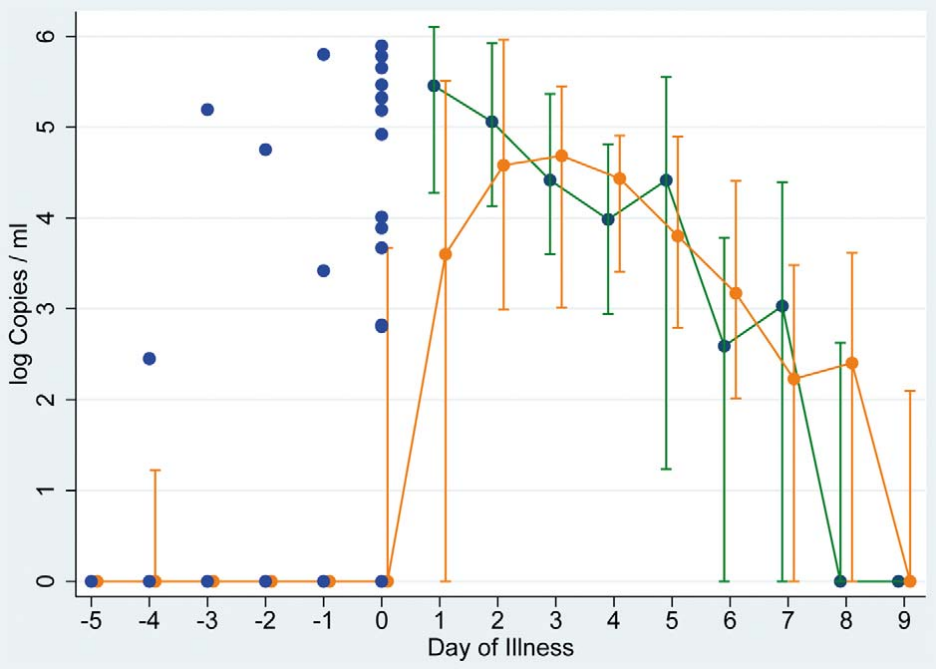
Viral shedding in laboratory confirmed symptomatic index and secondary cases. Median and interquartile range of log Copies/ml in index patients (green line) and secondary symptomatic cases (orange line) as well as individual values for participants with presymptomatic shedding (blue dots). For negative tests a value of 0 was used. By definition, symptom onset started on day 1.

Analysis of shedding curves among symptomatic influenza patients included data from 180 individuals (122 index patients, 58 household contacts). Figure 2 shows an analysis of the median viral load by illness day and includes all virus (sub)types and all laboratory confirmed cases, stratified for index and secondary cases. The shedding curve of index patients starts from a high point on the first illness day and declines from there. The number of copies detected in specimens from secondary cases starts on a lower level than those among index cases, but reaches a peak on illness day 3. From that time point both curves are largely the same. We detected presymptomatic shedding primarily on the first day before symptom onset. The proportion of influenza-positive presymptomatic samples was 30% (12/40), 10% (2/20), 9% (1/11), 20% (1/5), 25% (1/4) and 0% (0/3) on the first, second, third, fourth, fifth and sixth day before symptom onset, respectively (corresponding to illness days −1, −2, −3, −4 −5 and −6 (when the day before symptom onset is noted as day −1). Out of five individuals of whom samples beyond three days before symptom onset were available shedding was observed in one. This patient was infected with influenza A(H1N1)pdm09 and shed influenza virus on three out of five days prior to symptom onset. To illustrate presymptomatic shedding, we show individual viral load values before symptom onset in figure 2.

**Figure 3:**
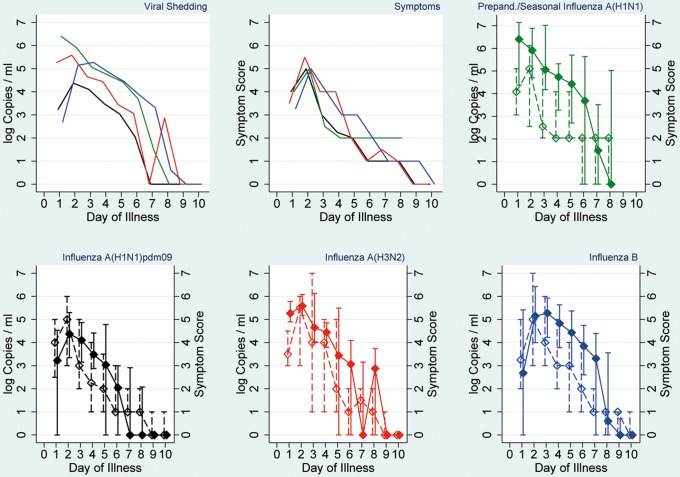
Viral shedding and symptom scores in patients with influenza-like illness symptoms. Viral shedding (expressed as median of log Copies/ml) and symptom scores (expressed as median) for all cases with influenza-like illness symptoms. Top left: viral shedding, top middle: symptom scores; top right: seasonal/prepandemic influenza A(H1N1), bottom left: influenza A(H1N1)pdm09, bottom middle: influenza A(H3N2), bottom right: influenza B. For negative tests a value of 0 was used.

Further analysis of shedding curves was done for influenza patients with ILI comprising 112 (92% of 122) of index and 42 (63% of 67) secondary cases. Shedding curves stratified by virus (sub)type are presented in figure 3. All (sub)types display a comparable shedding profile with the highest viral loads on illness days 1–3 followed by a steady decline. Median viral load of influenza A(H1N1)pdm09 was lower than that of the other (sub)types on days 3–6. Also noteworthy is the shedding curve of influenza B which starts from the lowest point, rises to it’s peak by day 2 and 3 and continues to have the highest viral load until day 7 (figure 3, upper panel, left). Symptom scores followed a comparable pattern as the shedding curves (figure 3, upper panel, middle) peaking on illness day 2 in all (sub)types. In contrast to the other (sub)types, however, the median of symptoms score for seasonal/prepandemic influenza A(H1N1) did not recede completely by the end of the participants’ observation period on illness day 8.

**Figure 4:**
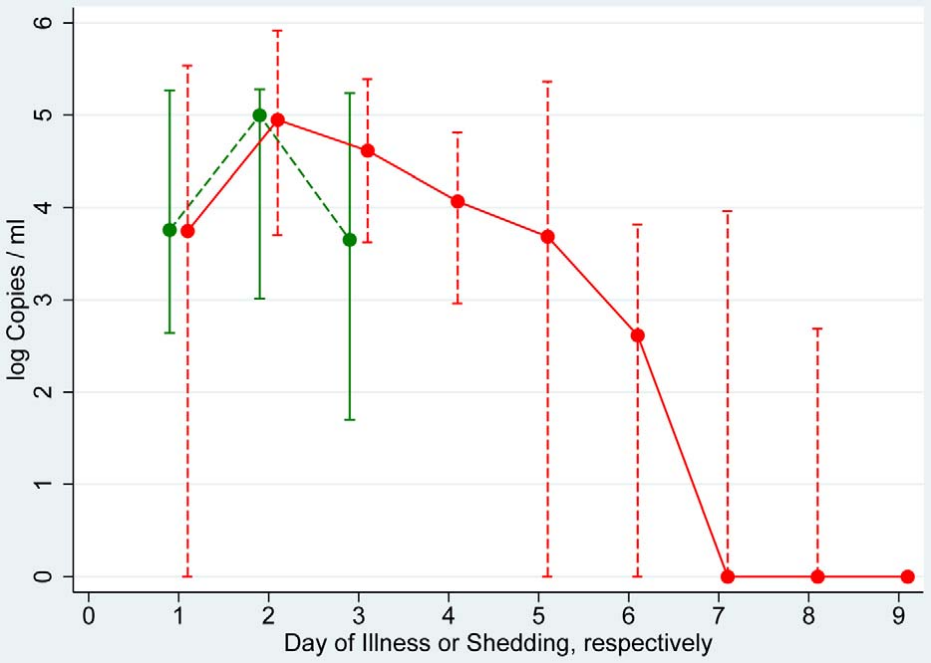
Symptomatic and asymptomatic shedding in influenza patients. Median and interquartile range of log Copies/ml in 6 asymptomatic influenza patients (green; 2 A(H1N1)pdm09, 2 A(H3N2), 2 B) as well as in influenza patients with influenza-like illness of any type or subtype (red).

Specimens from asymptomatic/subclinical individuals were available from 6 participants (2 with A(H1N1)pdm09, 2 with A(H3N2) and 2 with influenza B infections) on day 1, from 3 participants on day 2 and from 4 participants on day 3. The amount of shedding does not differ substantially from the values pooled from all influenza patients with ILI (Figure 4).

**Figure 5:**
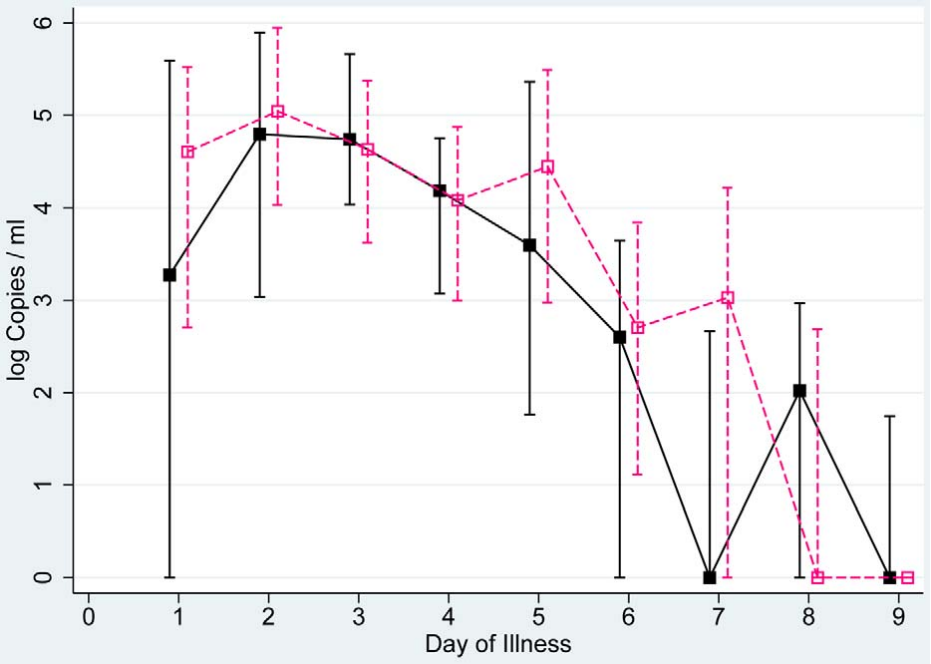
Viral shedding and symptom scores in patients with influenza-like illness stratified by age. Viral shedding (expressed as median and interquartile range of log Copies/ml) and symptom scores (expressed as median and interquartile range) for adult and child patients with influenza-like illness symptoms. adults - continuous line, children - dashed line) starting on the day of symptom onset. For negative tests a value of 0 was used.

Stratification by age (children vs. adults) showed similar profiles in influenza-positive ILI-patients for both groups until study day 6 (Figure 5). Differences between the two age groups were not statistically significant.

**Figure 6:**
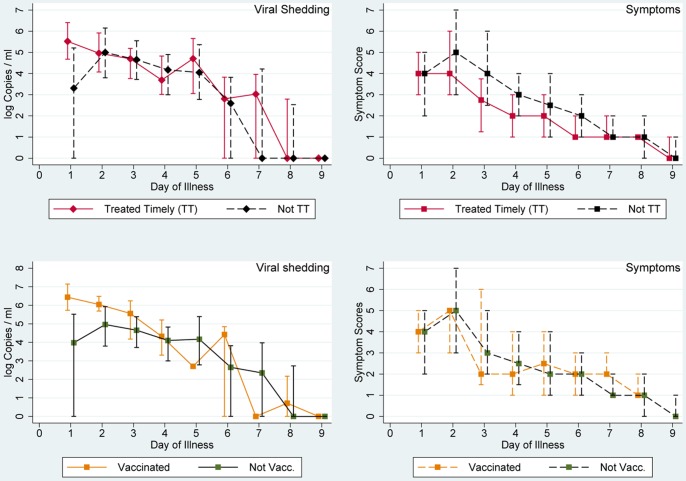
Influence of antiviral therapy and vaccination on viral shedding and symptoms in influenza patients. Viral shedding expressed as median and interquartile range of log Copies/ml and influenza symptoms expressed as median and interquartile range. Top: patients with influenza-like illness, by antiviral treatment, bottom: not vaccinated patients (all with influenza-like illness) in green, vaccinated patients (with any symptoms) in orange. For negative tests a value of 0 was used.

The effect of antiviral therapy on viral shedding and symptoms of influenza patients with ILI is shown in figure 6. The symptom curve suggests a milder clinical course and a slightly earlier resolution of symptoms in the treatment group (figure 6). We found significantly lower symptom scores in the group with timely treatment on illness days 3 (p = 0.0007) and 4 (p = 0.001).

Five index patients (three with influenza B, one each with seasonal/prepandemic influenza A(H1N1) and influenza A(H1N1)pdm09, respectively) and two secondary cases (both with influenza A(H1N1)pdm09) contracted influenza despite being vaccinated. Of 43 specimens collected from these vaccinated patients 27 (63%) tested positive by qRT-PCR, and of 1379 specimens from non-vaccinated cases 751 (55%) tested positive. The difference between these groups was not statistically significant. The viral shedding and the symptom profile of vaccinated cases) were comparable to those of non-vaccinated ILI cases (figure 6, lower panel, left).

**Figure 7:**
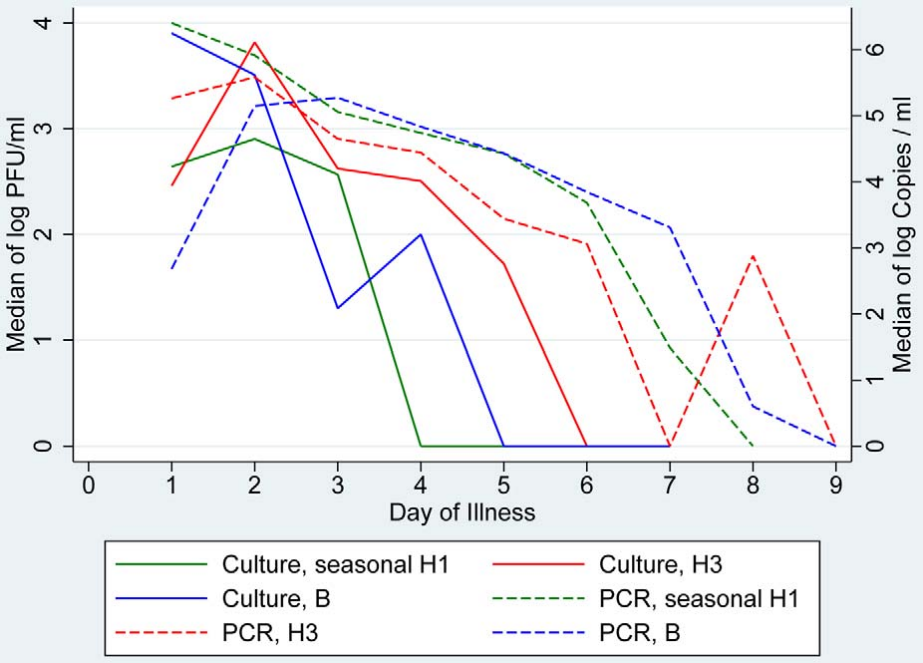
Viral load in specimens measured by qRT-PCR or viral culture, by (sub)type. Viral load in specimens of patients with influenza-like illness measured by qRT-PCR (expressed as median of log Copies/ml) as well as in patients with any symptoms measured by viral culture expressed as median of log Plaque forming units (PFU), stratified by (sub)type. No data available for influenza A(H1N1)pdm09. For negative tests a value of 0 was used.

The shedding profiles measured by viral culture for influenza viruses of (sub)types seasonal/prepandemic A(H1N1), A(H3N2) and influenza B (figure 7, continuous lines) compared with shedding profiles measured by qRT-PCR in ILI patients (figure 7, dashed lines) showed that the duration of viable virus was shorter for all examined (sub)types (seasonal/prepandemic influenza A(H1N1): 4 days (PCR: 8 days), influenza A(H3N2): 6 days (PCR: 7–9 days) and influenza B: 5 days (PCR: 9 days).

## Discussion

In this study, conducted in Germany during four consecutive influenza seasons (including the pandemic and first postpandemic wave), we provide important data on virologic and epidemiologic parameters of pre- and postpandemic influenza viruses, i.e. seasonal/prepandemic A(H1N1), pandemic (H1N1)pdm09, A(H3N2) and B. The household based approach to gather this type of information is an efficient and appropriate design to collect those kind of data in the natural setting. Advantages are that it permits identification and measurement of presymptomatic and asymptomatic shedding and that clinical symptoms of secondary cases can be described without surveillance bias because secondary household cases were identified through serial testing without the use of a case definition.

Overall 63% of non-vaccinated secondary household cases had an ILI-syndrome and the proportion of asymptomatic/subclinical secondary cases was 14%. Frequency distribution of clinical symptoms did not differ between A(H1N1)pdm09 cases and non-pandemic influenza cases. These observations are similar to results of other studies that analysed naturally occurring pandemic [12] or prepandemic/seasonal infections [6], [13]. However, we might have missed short-lived shedding in infected asymptomatic/subclinical participants, as we did not conduct additional serologic testing. Interestingly, 21% of adult secondary cases were asymptomatic/subclinical, while all children that contracted influenza were symptomatic. If replicated in other studies this finding may be important for public health measures as children are known to play an important role in the transmission of influenza, and high rates of asymptomatic/subclinical infection in this age group might diminish the probability for the success of potential prevention strategies.

Recent research has shown that serial intervals may be different in different settings, i.e. school, community or household [14]. From our household data we estimated the serial interval for A(H1N1)pdm09 to be 2.4 days which is similar to an estimate of 2.6 days derived from a review of thirteen studies [15]. The true serial interval may be different because we have discarded secondary household cases that occurred at least one day after the onset of the first secondary case in the household because we do not know if this case represents a secondary or tertiary case. For seasonal influenza viruses, our estimates were 2.4 days (seasonal/prepandemic A(H1N1)), 1.9 days A(H3N2) and 2.4 days (B), respectively. Two other publications on prepandemic influenza approximated the serial interval as 3.6 days (no (sub)type specified) [16] or as 2 days for A(H3N2) [17]. In a review of challenge studies conducted before the pandemic (H1N1) 2009, Carrat et al. computed the generation time of seasonal influenza viruses as 2.3 days (H1N1), 3.1 days (H3N2) and 3.4 days (B) [5]. Overall it appears that serial intervals are similarly short for all virus (sub)types. This supports the notion - based on the experience from controlled trials on the effect of non-pharmaceutical interventions - that any measures aiming to prevent (household) transmission must be implemented quickly to be effective [8], [18].

Published reports suggest that influenza RNA may be detected in blood at best sporadically [19]–[21]. We also were not able to demonstrate viraemia in selected cases of seasonal influenza. In contrast, one study conducted during the influenza pandemic (H1N1) 2009 detected viral RNA in the blood of 14 out of 139 patients hospitalised with influenza. However, viraemia was only seen in severely ill patients [22]. Influenza virus RNA (occasionally also viable virus) has been identified in stool samples of patients suffering from seasonal influenza [23]. Studies on A(H1N1)pdm09 [24], [25] have also been able to demonstrate viral RNA in stool samples from both community [24] and hospitalised patients [25]. The stool sample of one hospitalised child with a very high viral load even yielded a positive culture [26]. In our small collection of stool samples we did not identify viral RNA, perhaps because none of the participants was severely ill which might predispose to viral shedding in blood or feces.

Pooled shedding data of all four influenza virus (sub)types examined in this study showed presymptomatic shedding on the day preceeding symptom onset in 30% of participants providing samples on that day. This is consistent with a proportion of 27% for seasonal influenza A (H1N1 or H3N2) cases and 29% for influenza B cases as published by Lau et al. [13]. Presymptomatic shedding beyond the first day is difficult to interpret and might be explained by gradual disease onset or an exceptionally latency period in an individual case. Overall, published reports on presymptomatic shedding are rare, likely due to the methodological difficulties in obtaining data on this subject. Donnelly estimated that a substantial proportion (15–25%) of transmission of pandemic (H1N1)pdm09 occurs from viral shedding before disease onset [27]. This would be supported by our data although a direct comparison is not possible. Another study that investigated three case clusters very early in the pandemic (H1N1) 2009 came to the conclusion that transmission may have occurred during the presymptomatic phase [26]. Unfortunately, we cannot provide information on the question of disease transmission during presymptomatic shedding periods.

The degree of viral load associated with asymptomatic (or subclinical) infection could be assessed in 6 participants only and suggested that the amount of shedding may be similar to that in symptomatic influenza patients with ILI symptoms. This finding concurs with results from Loeb [7] and Lau [13]. While Loeb found that asymptomatic shedding lasted 4.0 days [7] our data did not suffice to determine the duration of shedding in asymptomatic/subclinical infections.

The shedding curves of the four virus (sub)types in ILI-patients do not show considerable differences, however two points can be made: (i) viral load of influenza A(H1N1)pdm09 was rather lower and shorter than in patients infected with seasonal (sub)types, its profile is similar to seasonal influenza A viruses with an early peak on illness days 1 or 2 and a steady decline thereafter (consistent with data from Cowling et al. [6]); (ii) upon close examination, the molecular shedding curve of influenza B starts from the lowest point, peaks by day 3 and continues to have the highest viral load of all viruses. Other publications on shedding of influenza B are scarce. Lau et al. also based their investigations on naturally occurring infections and found, similar to us, a peak for seasonal A viruses on days 1 or 2, whereas the shedding pattern of influenza B was delayed to day 3 [13]. Also the influenza B shedding curve (based on a single study) presented in the review by Carrat et al. [5] demonstrates a slow increase with a peak on the 4^th^ day after inoculation, corresponding approximately to illness day 3. Thus, molecular viral shedding of both seasonal and pandemic influenza A viruses may peak one or two days earlier than influenza B.

In contrast, symptom curves of ILI patients with infections of all four (sub)types appear to follow a very similar course (figure 3, top panel, middle picture). All peak on day 2 and decrease steadily thereafter until illness days 8 or 9. Again, other investigators have reported similar findings [6], [13] specifying that systemic signs, such as fever, subside first, followed by symptoms of the lower and finally of the upper respiratory tract.

Based on the population of ambulatory patients investigated we found no evidence that the amount of shedding is particularly higher in children, nor that duration of viral shedding is significantly longer in children compared to adults. A few smaller studies on influenza (H1N1)pdm09 have also not been able to show significant age-dependent differences in the duration of viral shedding [28], [29], although others, both in seasonal [30] and pandemic influenza A(H1N1)pdm09 [31], have. The latter study was conducted among patients hospitalised with A(H1N1)pdm09 and demonstrated very clearly that duration of shedding is prolonged in hospitalised children <13 years [31]. While all of our patients had infections that were mild enough to be treated on an out-patient basis, it is possible that an age difference in viral shedding becomes particularly visible when the course of disease is severe.

In 1997, a randomized controlled trial with experimental infection of human volunteers demonstrated that very early administration of oseltamivir (in this case 28 hours after inoculation, i.e. approximately at the time of symptom onset) can reduce viral shedding and significantly ameliorate the clinical course [32]. Two randomized trials where influenza patients with naturally acquired infection were treated with oseltamivir no later than 36 hours after symptom onset also showed a significant reduction of severity, but differed in their assessment of the effect on viral shedding [33], [34]. Observational studies where requirements for oseltamivir treatment permitted treatment inception up to 48 hours after symptom onset often failed to demonstrate significant effects in viral shedding parameters [6], [28], [29] and our study is no exception in this regard.

To our knowledge, the effect of vaccination on viral shedding among patients who have become infected despite having been vaccinated has been investigated rarely.

We identified seven influenza-positive patients who had been vaccinated: two symptomatic household contacts and five index patients. We were surprised to observe that both the proportion of positive samples as well as the viral load among vaccinated cases was similar compared to that among non-vaccinated cases. The only study known to us where these questions have been studied [35] reported similar results. Also this finding may have important implications for the parameterisation of modelling studies. Caution should be taken to avoid misinterpretation, though. It does not mean that the vaccine is not effective. Of 67 vaccinated household contacts only two (3%) contracted influenza. Similarly, in the cited publication by Couch et al., vaccine efficacy for symptomatic illness was 93% and for any shedding 70%, but the probability of being asymptomatic among those who shed was 86% (6/7) in vaccinated and 45% (5/11) in non-vaccinated.

To compare molecular data on viral load with viral culture we directly cultured viruses from the seasonal/prepandemic (sub)types A(H1N1), A(H3N2) and B. Cultures of A(H1N1)pdm09 were not done. We observed that samples from illness days 4–6 cease to contain viable virus, but viral RNA may still be detectable until illness days 7–9. This compares well with data from seasonal influenza obtained from an experimental study in volunteers where the duration of shedding in untreated adult volunteers using viral culture was found to be 4.8 days [5].

There are some limitations which need to be considered in the context of this study. As recruitment of index patients was based on rapid antigen testing and an increased level of viral shedding is associated with increased rapid test sensitivity [36], our data on viral shedding and the course of illness may represent (likely more severe) infections associated with generally increased levels of shedding. In addition, most analyses of shedding characteristics were done among ILI patients which are likely not representative of all influenza patients. For logistic reasons, viral culture could only be performed during seasons 1 and 2. Thus sample sizes were small and comparability with other studies is limited. Furthermore, sample collection differed slightly between seasons 1/2 and 3/4 with household visits scheduled every day during the first two and every second day during the second two seasons. There is a slight chance that we may have missed secondary, very low-grade and/or short-lived, asymptomatic/subclinical infections between these visits. Overall, the quantity of data and hence statistical power was limited, so we may not have found differences that may in fact exist.

The strengths of our study are (i) data collection from naturally occurring infections over the course of four seasons with circulation of four different (sub)types, (ii) prospective identification, collection of specimens and questionnaire data from study participants, which enabled us to obtain a true overall clinical picture of influenza cases.

In summary, our study addresses several important questions on clinical manifestation, duration of infectiousness, viral shedding patterns, including shedding before symptom onset and in asymptomatic/subclinical patients, as well as the effect of vaccination and antiviral therapy on viral shedding. Important single results include the finding that children do not seem to be infected asymptomatically, that shedding one day before symptom onset may occur in one third of influenza patients, that asymptomatic/subclinical influenza patients occur rarely, but viral load (and probably infectiousness) may be substantial, and vaccinated influenza patients do not show different shedding patterns compared to non-vaccinated cases with ILI. Overall results do not show marked differences between seasonal influenza (sub)types and influenza A(H1N1)pdm09.
